# Fibro-Bronchitis and Rheumatic Pneumonia

**Published:** 1854-02

**Authors:** 


*Fibro-Bronchitis and Rheumatic Pneumonia.* By Thomas H. Bucklor, M. D. etc. Philadelphia: Blanchard & Lea.

THE author describes a fibrous or rheumatic inflammation of the bronchial tubes separate and distinct from inflammation of the mucous membrane.

The first part of the essay consists in an enunciation of the authors position together with the authorities, especially the French, the quotations from which would have been more acceptable to most readers, had they been translated. The second part consists in reports and analyses of cases, together with the treatment.

The author has evidently studied the subject carefully and thoroughly, and his book will be read with interest.

For sale by Cooke and Co. J.

